# Transcriptional activation of microRNA-34a by NF-kappa B in human esophageal cancer cells

**DOI:** 10.1186/1471-2199-13-4

**Published:** 2012-01-31

**Authors:** Juan Li, Kai Wang, Xuedan Chen, Hui Meng, Min Song, Yan Wang, Xueqing Xu, Yun Bai

**Affiliations:** 1 Department of Medical Genetics, College of Basic Medicine, Third Military Medical University, Chongqing, People's Republic of China

**Keywords:** miR-34a, NF-kappa B, p53, gene expression regulation

## Abstract

**Background:**

miR-34a functions as an important tumor suppressor during the process of carcinogenesis. However, the mechanism of miR-34a dysregulation in human malignancies has not been well elucidated. Our study aimed to further investigate the regulation mechanism of miR-34a.

**Results:**

We found that overexpression of NF-kappa B p65 subunit could increase miR-34a levels in EC109, an esophageal squamous cancer cell line, while ectopic expression of DN IkappaB leaded to a significant reduction of miR-34a expression. Bioinformatics analysis suggested three putative KB sites in promoter region of miR-34a gene. Mutation two of these KB sites impaired p65 induced miR-34a transcriptional activity. Chromatin immunoprecipitation and electrophoretic mobility shift assays both showed that NF-kappaB could specifically bind to the third KB site located in miR-34a promoter. In addition, we found that overexpression of NF-kappaB p65 could not successfully induce miR-34a expression in esophageal cancer cell lines with mutant p53 or decreased p53. Reporter assay further showed that NF-kappaB-induced miR-34a transcriptional activity was reduced by p53 impairment. Nevertheless, CHIP analysis suggested binding of NF-kappaB to miR-34a promoter was not affected in cells with mutant p53.

**Conclusions:**

Our work indicates a novel mechanism of miR-34a regulation that NF-kappaB could elevate miR-34a expression levels through directly binding to its promoter. And wildtype p53 is responsible for NF-kappaB-mediated miR-34a transcriptional activity but not for NF-kappaB binding. These findings might be helpful in understanding miR-34a abnormality in human malignancies and open new perspectives for the roles of miR-34a and NF-kappaB in tumor progression.

## Background

MicroRNAs (miRNAs) are small, non-coding RNAs that negatively regulate gene expression at the posttranscriptional level. Emerging evidence has demonstrated that these small RNAs are involved in almost every aspects of tumor biology and could function as oncogenes or tumor suppressor genes [1]. MiR-34a has recently been found to act as an important tumor suppressor in the development of various cancers. A variety of genes referring to cell cycle and apoptosis control, such as CDK4/6, cyclin D1, E2F3, MYCN, and SIRT1and Bcl2 are demonstrated to be downregulated by miR-34a [2-11]. MiR-34a could also inhibit cell migration and invasion through targeting c-Met [12]. Recent data suggest that dysregulation of miR-34a exists in many types of human cancers and is correlated with clinic treatment [13-17]. Although the most important regulator of miR-34a expression is the well-known tumor suppressor p53 [7-10], p53 abnormality is not always correlated with low levels of miR-34a in human cancer tissues. In chronic lymphocytic leukemia, deletion or mutation of p53 is associated with miR-34a downregulation [14-16]. While in neuroblastoma and non-small-cell lung cancer, no significant correlation between p53 mutation and miR-34a dysregulation is observed [17]. Though researchers have reported other mechanisms for miR-34a abnormality, like deletion of 1p36.3, aberrant CpG methylation or CEBPα mutation [13,17-19], more detailed study on regulation of miR-34a transcription is of great importance.

Nuclear factor-kappa B (NF-κB) is a ubiquitously existed transcription factor regulating expression of numerous genes involving in inflammation, immune and cancer progression [20]. In an inactive status, NF-κB family members exist as dimmers with the predominance of p65/p50 heterodimers and are sequestered in the cytoplasm by members of IκB family. When NF-κB pathway is activated by a series of stimuli, IκB proteins are phosphorylated and degraded in a proteasome dependent manner leading to nuclear translocation of NF-κB and activation of downstream gene expression [21]. Although numerous studies suggest that activation of NF-κB signal lead to resistance to apoptosis, incontrollable cell proliferation, metastasis, and angiogenesis [20], there are also evidence indicating a tumor-suppressor like effect of NF-κB. Different research groups have independently reported that blockade of NF-κB signal pathway caused a significant increase in spontaneous epithelial squamous cell carcinoma or diethylnitrosamine induced hepatocellular carcinoma [22-24]. And also expressions of several proapoptotic genes, such as Fas, Puma and DR5 have been demonstrated to be upregulated by NF-κB [25-27]. Thus, we hypothesized that NF-κB might have the ability of regulating expression of tumor suppressive miRNAs. Interestingly, bioinformatics analysis revealed that there were several potential NF-κB binding sites located in the promoter region of miR-34a gene. So, we wondered if miR-34a was a direct target of NF-κB transcription factor. And this study was aimed to investigate whether NF-κB could directly regulate miR-34a expression and the way how they regulated.

## Results

### NF-κB activation correlates with miR-34a expression

**Figure 1:**
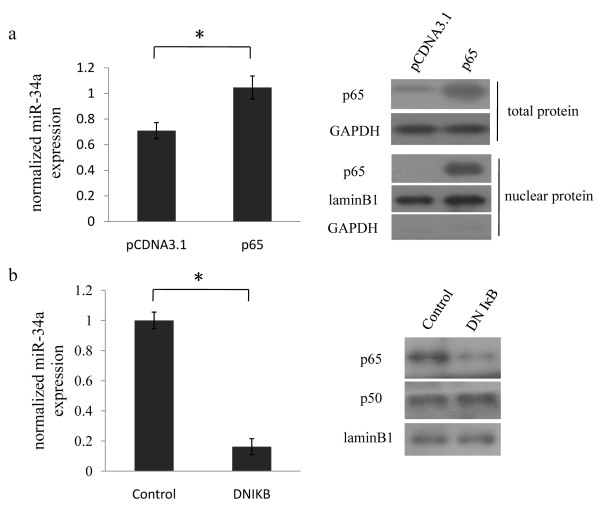
**NF-κB activation correlates with miR-34a expression**. (a) EC109 cells were transfected either with empty vector (pcDNA3.1) or p65 expression vector (p65), 48 h after transfection, changes of total p65, nuclear p65 and mature miR-34a levels were individually detected by western blot or qRT-PCR. (b) EC109 cells were transfected either with empty vector (control) or with dominant negative mutant of IκB (DN IκB), nuclear p65 and p50 protein levels and mature miR-34a levels were individually detected by western blot or qRT-PCR.

In order to study whether NF-κB could regulate miR-34a expression, two different approaches were used to alter NF-κB function in EC109 cells, an esophageal squamous cancer cell line containing wildtype p53 (data not shown). First, we enhanced the NF-κB p65 levels by transfecting EC109 cells with a p65 expressing vector, cells with overexpressed p65 exhibited an increase of miR-34a levels compared with the vector control cells (Figure 1a). Next, we blockaded the endogenous NF-κB activity by transfecting EC109 cells with dominant negative IκBα which was refractory to phosphorylation mediated degradation. Transfection of this mutant IκBα indeed leaded a significant reduction of nuclear NF-κB p65 translocation. And the expression of miR-34a was also sharply decreased in cells lacking NF-κB activity (Figure 1b). These results suggested that NF-κB activation was needed for miR-34a expression and activation of NF-κB signal could upregulate its expression levels.

### Overexpression of NF-κB p65 subunit enhances the transcriptional activity of miR-34a

**Figure 2:**
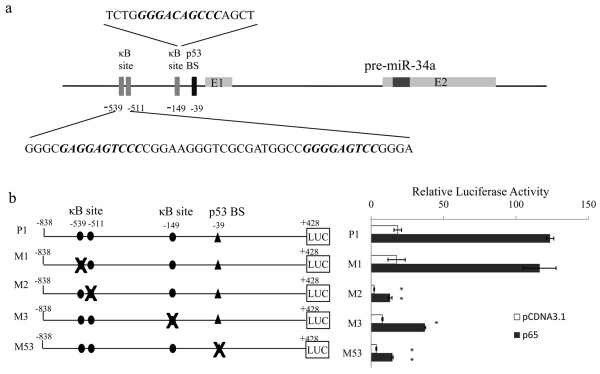
**Overexpression of NF-κB p65 subunit enhances the transcriptional activity of miR-34a**. (a) Genomic structure for human miR-34a gene. (b) EC109 cells were transfected with pCDNA3.1 or p65 in combination with the wildtype miR-34a promoter (P1) or promoter with different NF-κB binding site mutated (M1-M3) or promoter with p53 binding site mutated (M53). pRL-TK reporter plasmid was used as transfection control. 48 h later, cells were lysised and luciferase assay were performed as described in Materials and Methods. Error bars represent the standard deviations for three independent experiments. *P < 0.05 * * P < 0.01, t-test versus the P1 reporter vector control.

According to the bioinformatics analysis, three putative NF-κB binding sites were existed in miR-34a promoter region, located upstream of the known p53 bind site (Figure 2a). To further study whether miR-34a was transcriptionally regulated by NF-κB, about 1.3 kb DNA fragment containing the three putative NF-κB binding sites and the known p53 binding site was cloned into a promoterless luciferase reporter vector (P1). Also we constructed another four reporter vectors with different κB site mutated (M1, M2, M3) or p53 binding site mutated (M53) (Figure 2b). Cotransfected p65 subunit with the wildtype reporter vector into EC109 resulted in a markable enhancement of luciferase activity compared with the control. Mutation of the first NF-κB binding site had no impact on the transcriptional activity enhancement caused by p65 overexpression, while mutation of the other two greatly weakened the transactivity ability (Figure 2b). These indicated that these two sites had critical roles in transcriptional regulation of miR-34a by NF-κB. Furthermore, we also found that mutation of the p53 binding site in miR-34a promoter region impaired the transcriptional enhancement mediated by p65, suggesting p53 may involve in NF-κB mediate induction of miR-34a.

### NF-κB directly binds to the promoter region of miR-34a gene

**Figure 3:**
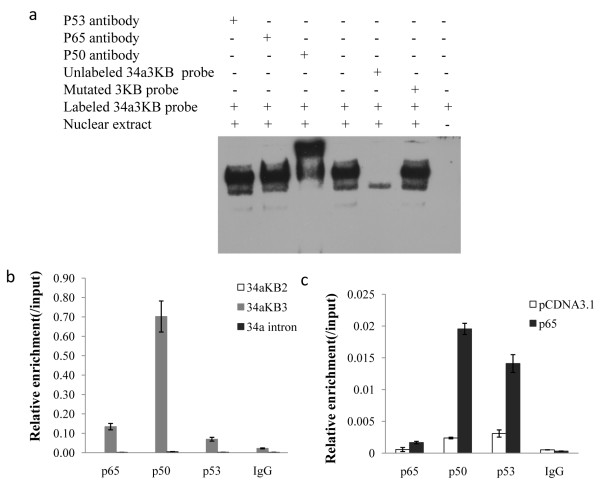
**NF-κB directly binds to the promoter region of miR-34a gene**. (a) EMSA was performed with EC109 cells nuclear extracts using probe corresponding to the third κB site (34a3ΚB) in miR-34a promoter region. Competition and supershift assays against p53, p65 and p50 were also performed. (b) Chromatin derived from EC109 cells were immunoprecipitated with anti-p65, p50, p53 and IgG antibodies. Relative enrichment of each transcription factor-bound DNA was detected by qPCR using primers described in the method. Shown were results normalizing to input DNA. (c) CHIP assay were performed with EC109 cells transfected with p65 or control vectors, Relative enrichment of each transcription factor-bound DNA was detected by qPCR using primer 34aΚB3.

Since NF-κB could regulate miR-34a expression at the transcriptional level, we next investigated whether NF-κB could directly bind to the promoter region. EMSA assay was first performed. Two oligonucleotides were used in the experiment: 34a2KB probe containing the second κB site and 34a3ΚB containing the third κB site. 34a3ΚB probes were detected bound with protein of nuclear extract from EC109 cells, and supershift was appeared in the presence of anti-p50 antibody (Figure 3a). No specific binding by nuclear extract protein was observed with 34a2ΚB (data not shown). These results suggested that regulatory region of miR-34a gene could be bound with NF-κB dimmers in EC109 cells.

Then we performed chromatin immunoprecipitation analysis with EC109 cells to detect the binding of NF-κB in vivo. Three PCR amplicons were designed: one flanking the first two κB sites in miR-34a promoter region (34aKB2), another flanking the third κB site (34aKB3) and the last one located in the intronic region of miR-34a gene served as a control (34a intron). As shown in Figure 3b, recruitment of p65 and p50 NF-κB subunit to the promoter region containing the third κB site was observed in EC109 cells. However, promoter region flanking the first two κB sites and the intronic region had no obvious enrichment of these transcription factors. Furthermore, binding of these transcription factors in EC109 cells transfected with p65 were also detected. As shown in Figure 3c, binding of NF-κB p65 and p50 subunits to the promoter region containing the third κB site were boosted in cells with overexpressed p65. No obvious enrichment in the first two κB sites was detected in transfected cells (data not shown). Enhanced p53 binding to miR-34a promoter was observed too. These results demonstrated that increased binding of NF-κB to miR-34a promoter was indeed responsible for the induction in those transfected cells.

### P53 is necessary for NF-κB induced miR-34a transcription but not for NF-κB binding

**Figure 4:**
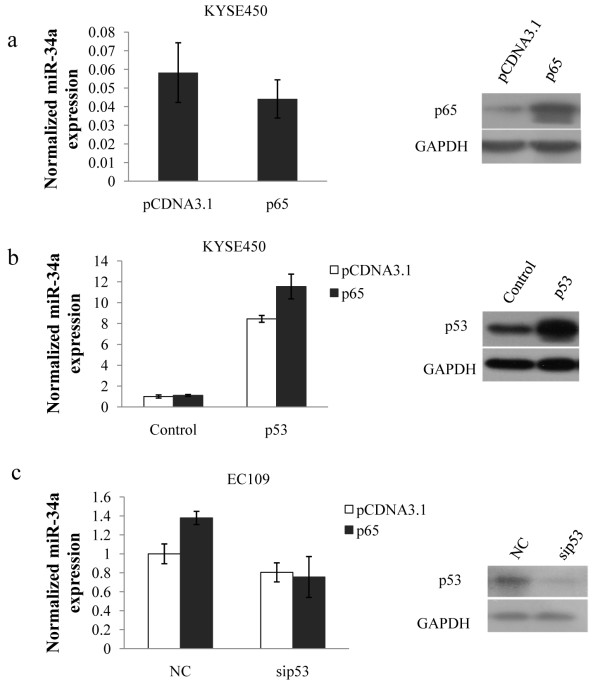
**P53 is required for NF-κB-mediated miR-34a induction**. (a)KYSE450 cells were transfected either with empty vector (pcDNA3.1) or with p65 expression vector (p65), 48 h after transfection, levels of p65 and mature miR-34a were individually detected by western blot or qRT-PCR. (b)KYSE450 cells were cotransfected with wildtype p53 or control vectors and p65 expression vectors or pCDNA3.1, 48 h after transfection, levels of p53 and mature miR-34a were individually detected by western blot or qRT-PCR. (c) pCDNA3.1or p65 and siRNA control or sip53 were cotransfected into EC109 cells. P53 protein and mature miR-34a levels were individually detected by western blot or qRT-PCR 48 h after transfection.

For mutation of the known p53 binding site could attenuate the effect of p65 on miR-34a transcriptional activity, we speculated that p53 may coordinate with NF-κB to regulate miR-34a transcription. In order to demonstrate this, KYSE450, another esophageal squamous cancer cell line containing a mutant form of p53 (p53^H179R^) was used in the following experiments. First, we transfected KYSE450 cells with p65 expression vectors, and ectopic p65 expression could not successfully induced miR-34a expression (Figure 4a). When reintroducing wildtype p53 into KYSE450 cells, miR-34a induction by p65 overexpression was observed (Figure 4b). In addition, we decreased p53 protein levels in EC109 cells using siRNA, and found that p65 was unable to upregulate miR-34a expression then (Figure 4c). These findings gave us a hint that p53 was required for NF-κB-mediated miR-34a induction and promoted us wondering how p53 affect this regulation.

**Figure 5:**
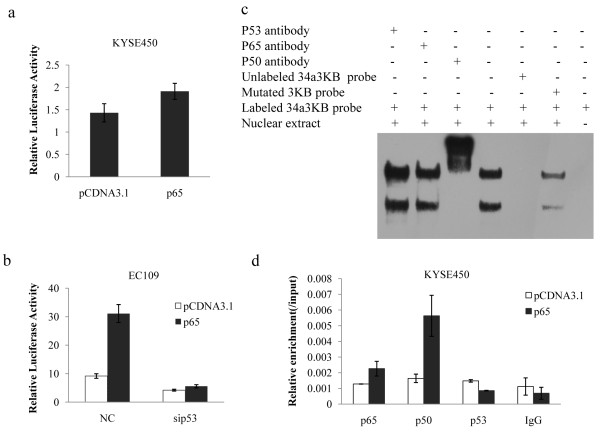
**P53 is necessary for NF-κB-induced miR-34a transcriptional activity but not for NF-κB binding**. (a) KYSE450 cells were transfected with pCDNA3.1or p65 in combination with the wildtype miR-34a promoter (P1). pRL-TK was used as transfection control. Cells were lysised and luciferase assay were performed 48 h after transfection. Error bars represent the standard deviations for three independent experiments. (b) pCDNA3.1or p65 and siRNA control or sip53 along with the wildtype miR-34a promoter were cotransfected in EC109. pRL-TK vectors were used as transfection control. Luciferase assay were performed 48 h after transfection. Error bars represent the standard deviations for three independent experiments. (c) EMSA was performed with nuclear extracts from KYSE450 cells using probe corresponding to the third κB site (34a3ΚB) in miR-34a promoter region. Competition and supershift assays against anti-p53, p65 and p50 antibody were also shown. (d) Chromatin derived from KYSE450 cells transfected with p65 or control vectors were immunoprecipitated with anti-p65, p50, p53 and IgG antibodies. Relative enrichment of each transcription factor-bound DNA was detected by qPCR using 34aΚB3 primers. Shown were results normalizing to input DNA.

We then performed luciferase reporter assay in KYSE450 cells and EC109 cells with decreased p53 levels. Unlike the performance in normal EC109 cells, cotransfection of p65 with miR-34a promoter reporter vectors (P1) only cause a slight increase of the luciferase activity in KYSE450 cells(Figure 5a). And knock-down of p53 level dramatically impaired the transactivity enhancement caused by p65 (Figure 5b). These indicated that impairment or loss of wildtype p53 function might affect NF-κB p65-induced transactivity of miR-34a. We next determined the binding of NF-κB subunits in KYSE450 cells. Surprisingly, nuclear extracts from KYSE450 cells could still bound with 34a3KB probes containing the third κB site of miR-34a promoter (Figure 5c). CHIP assay performed with transfected KYSE450 cells also showed an enhancement of NF-κB binding but not p53 binding (Figure 5d). These findings suggested that NF-κB binding to miR-34a promoter seems not be affected by loss of wildtype p53 function.

**Figure 6:**
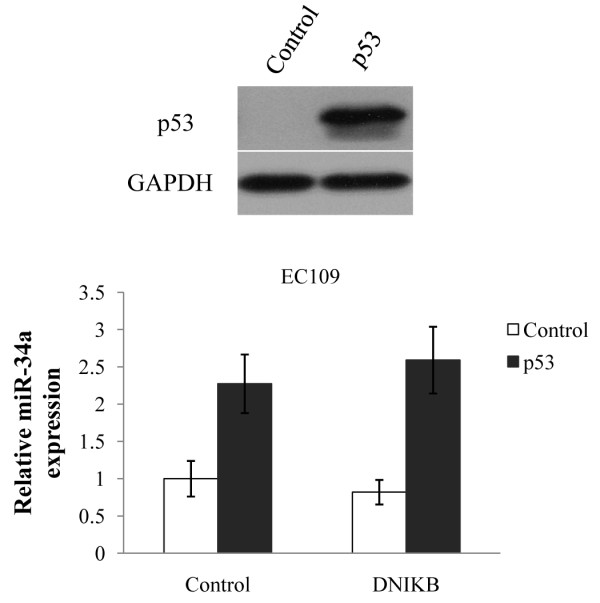
**Blocking of NF-κB activation might not affect miR-34a induction by p53**. EC109 cells were cotransfected by wildtype p53 or control vectors and DNIκB or control, p53 protein and mature miR-34a levels were individually detected by western blot or qRT-PCR 48 h after transfection.

The above results suggested NF-κB could regulate miR-34a expression. Since miR-34a is a known target of p53, we wondered if miR-34a induction by p53 required NF-κB. EC109 cells were cotransfected with DNIκB and wildtype p53, however, miR-34a levels were still increased by p53 overexpression in cells with decreased NF-κB activity (Figure 6). This suggested that NF-κB might not be necessary for p53-mediated miR-34a induction.

## Discussion

MiR-34a is an important tumor suppressive microRNA, which dysregulated in many types of cancers. Delicated study on its regulation mechanism is important for exploring new strategies for cancer therapy. In this study, we identified that:1) overexpression of NF-κB p65 subunit could increase miR-34a levels and ectopic expression of DN IκB leaded a significant reduction of miR-34a expression; 2) mutation of either the κB sites or the p53 binding site of miR-34a gene could impair p65-induced transcriptional activity; 3) NF-κB could specifically bind to the κB site located at -149 of miR-34a gene; 4) Expression of miR-34a could not be induced by NF-κB in the absence of wildtype p53 function, probably owing to the downregulated transcriptional activity; 5) NF-κB could bind with miR-34a promoter even in cells with mutant p53; 6) NF-κB might not be necessary for p53-mediated miR-34a upregulation. According to these findings, we concluded that NF-κB could directly activate miR-34a expression at the transcriptional level and wildtype p53 might be responsible for the transactivity but not for NF-κB binding.

Previous studies have demonstrated that miR-34a is a direct target of p53, our study revealed a novel mechanism for miR-34a regulation. NF-κB is an important transcript factor linking inflammation and immunity to cancer initial and progression. It could be activated by a variety of inflammatory cytokines existing in local environment of tumors [28]. Functioning as a downstream target of NF-κB, it is possible that miR-34a also involve in inflammation-related tumorigenesis. Surprisingly, Elodie Roggli has recently reported that miR-34a is indeed induced by two important inflammatory cytokines, IL-1β and TNFα, in human islet cells [29]. Thus, our study indicated that besides participating in p53 pathway, miR-34a might play a role in tumor microenvironment network through regulation by NFKB signal pathway. However, the delicated function needs further investigation.

Unlike many other transcription factors involved in cancer biology, NF-κB played a two-side role during the process of carcinogenesis. Downstream genes responsible for the tumor-promoting role of NF-κB have been studied exhaustively, such as antiapoptic gene, c-FLIP, Bcl-XL and IAP family member [30-32]; cell cycle regulator, cyclin D1 [33] and genes referring to environmental modification, vascular endothelial growth factors (VEGF) [34] and matrix metalloproteinases (MMPs)[35]. However, mechanism of tumor-suppressor role for NF-κB remains poorly understood. NF-kB had been reported to induce G1 cell cycle arrest in human epithelial cells through increasing of p21^cip ^or suppression of CDK4 [36,37]. Our work demonstrated miR-34a was a direct target of NF-κB. And miR-34a was verified to be able to induce cell growth arrest or apoptosis by downregulating expression of a variety of cell cycle regulator and antiapoptotic genes, including CDK4 [2-11,38]. Thus, our study also revealed a potential target for NF-κB responsible to its inhibitory role in cancer progression.

In this study, we found that NF-κB-mediated miR-34a induction required wildtype p53 function. Actually, previous studies have also demonstrated p53 might play a role in NF-κB mediated gene expression. For example, transcriptional activation of DR5 needed both NF-κB and p53 binding to the related sites and knock-down of p53 expression blocked the binding of p65 with DR5 gene. [27]. And also NF-κB has been reported to involve in gene transcription regulated by p53. For example, NF-κB p52 subunit could modulate several p53 downstream genes transcription through binding to their promoter region [39]. Our study revealed that NF-κB-mediated miR-34a transactivity might be affected by p53, but binding of NF-κB to miR-34a promoter was independent of p53. In addition, blocking nuclear translocation of NF-κB p65/p50 dimmers seemed to have no effect on p53-induced miR-34a expression.

## Conclusions

Our study demonstrates a novel mechanism of miR-34a regulation in human malignancies that NF-κB could regulate miR-34a expression. This is important for understanding the dysregulation of miR-34a in human cancer tissues and opens new perspectives for the function of miR-34a and NF-κB in tumor progression.

## Methods

### Plasmid construction

DNA sequence upstream of the human miR-34a gene was amplified by PCR using the following primer sets: 34aF CTGCTCGAGTGCCGGTTCCTGGCTTTA, 34aR GCGAAGCTTGCTGCAATATCACCGTG. PCR product was cloned into pGL3-basic vector (Promega) between the HindIII and XhoI sites. Site directed mutagenesis was performed by overlap extension PCR as described in [40]. And primers used were: 34aM53F TGCCTGGGTTTACCTGGGTTTATTCCGAGCCG, 34aM53R CGGCTCGGAATAAACCCAGGTAAACCCAGGCA; 34aM1F GGCGACGAGTCGCCGGAAGGGTCGCGAT, 34aM1R TTCCGGCGACTCGTCGCCCCTTCGCGGT; 34a M2F ATGGCCCGGGAGTCGGGGACCTCGGCTC, 34aM2R AGGTCCCCGACTCCCGGGCCATCGCGAC; 34a M3F TCGGTCTGGCGACAGCGCAGCTCCCCGGAT, 34a M3R AGCTGCGCTGTCGCCAGACCGACGGGAC. All the constructs were verified by sequencing.

NF-κB p65 and mutant IκB expression vector were kindly provided by Dr M. Cippitelli (University of Rome Sapienza, Rome, Italy) and Dr D. Fruci (Research Center, Ospedale Bambino Gesù, Rome, Italy). Wildtype p53 expression vector was a gift from Prof. Dong Wang (Third Military Medical University, Chongqing, China).

### Cell culture and transfection

Human esophageal cancer cell line EC109 were purchased from Cell Bank of Chinese Academy of Sciences, Shanghai, China. KYSE450 were obtained from Cancer Institute, Chinese Academy of Medical Sciences, Beijing, China. All cells were cultured in 1640 supplemented with 10% FBS, at 37°C in a humidified incubator containing 5% CO2.

For gene expression transfection, plasmids were transfected with lipofectamine2000 (Invitrogen) according to the manufacturer's instructions. Gene expression levels were detected 48 h after transfection.

### RNA extraction and Real-Time qRT-PCR

Total RNA was extracted using RNAiso reagent (Takara) according to the manufacturer's instructions and quantified with a NanoDrop spectrophotometer (Thermo Scientific). MiR-34a expression was measured using a TaqMan MicroRNA RT-PCR assay (Applied Biosystems). 100 ng total RNA was converted to cDNA using specific primers, and amplification of the cDNA was done using Taqman Universal PCR Master Mix (Applied Biosystems). PCR conditions were 95°C for 3 minutes followed by 40 cycles of 95°C for 15 seconds, 60°C for 40 seconds. The expression of MiR-34a was normalized against U6 snRNA expression.

### Western blot

For total protein analysis, cells were harvested and lysised in T-PER Tissue Protein Extraction Reagent (Pierce Chemical Company) with freshly added PMSF. For nuclear protein analysis, cells were lysised with Nuclear and Cytoplasmic Protein Extraction Kit (beyotime). Proteins were separated by SDS-PAGE and electrotransferred to PVDF membranes, after blocked with 5% skimmed milk, membranes were incubated with a primary antibody and then incubated with a horseradish peroxidase-conjugated secondary antibody. Antibodies used were anti- NF-κB p65 (sc-372, Santa Cruz Biotechnology), anti- NF-κB p50 (06-886, upstate), anti-p53 (sc-126x, Santa Cruz Biotechnology), anti-lamin B1 (SC-56145, Santa Cruz Biotechnology), HRP-conjugated monoclonal mouse anti-glyceraldehyde-3-phosphate Dehydrogease (KC5G5, Kangchen Bio-Tech) and horseradish-peroxidase coupled goat antibodies against rabbit and mouse immunoglobulins (Beijing Zhongshan Golden Bridge Biotechnology). The immunoreactive proteins were detected using SuperSignal West Dura Extended Duration Substrate (Thermo Scientific).

### Reporter assay

For reporter assay, p65 expression vectors or the control vectors were cotransfected with pGL3 reporter vectors along with pRL-TK vector (Renilla luciferase, Promega) using lipofectamine2000. Cell lysate was collected 48 h after transfection and luciferase activities were measured using Dual-Luciferase Reporter Assay System (promega). Activity was defined as Firefly/Renilla ratio and normalized to the negative control vector transfection.

### Chromatin Immunoprecipitation

ChIP was performed with a commercially available Chromatin Immuno-precipitation Kit (Upstate Biotechnology) according to the manufacturer's instructions. Briefly, cells (2 × 106/immunoprecipitation) were cross-linked in 1% formaldehyde for 10 min at room temperature and halted the cross-link with 0.125M glycine. Chromatin was captured with the following primary antibody: p65 (cell signal technology, 3987), p50 (upstate, 06-886), p53 (santa sc-126x), IgG (millipore, 12-371B). After overnight capture at 4°C, chromatin was collected, purified and then decrosslinked at 65°C. DNA was recovered using Spin Columns and the enrichment was detected by qPCR. Following primers were used in PCR assay: 34aΚB2, F CCCCCGTGGTTTCTGTTTG, R CCTGGGCTGGCGTTTC; 34aΚB3, F TGCGTGGTCACCGAGAAGCAG, R TTCAGGTGGAGGAGATGCCGC; 34a intron, F GCTCCATCCTCGGACCTGA, R GGCGGTCTGAGTTGGCTAG.

### Electrophoretic mobility shift assays

EMSA was performed according to the manufacturer's instruction of (Pierce). Nuclear protein of EC109 and KYSE450 cells were extracted using the Nuclear and Cytoplasmic Protein Extraction Kit (beyotime). In brief, biotin labelled DNA probes containing candidate NF-κB binding sites from miR-34a promoter (34aΚB2 TCGCGATGGCCGGGGAGTCCGGGACCTCGGCT, 34aΚB3 CCGTCGGTCTGGGGACAGCCCAGCTCCCCGGA, only one stand shown) were mixed with 10 ug of nuclear extract in a 20 ul reaction volume containing 1X binding buffer, 5 mM MgCl2, 5% glycerol, 0.05% NP40 and 50 ng/ul poly(dI:dC). The reaction mixture was incubated on ice for 30 min and applied to a 6% nondenatured polyacrylamide gel containing 0.5X TBE buffer. For competition assays, a 200-fold molar excess of unlabeled probes and unlabeled mutated probes (34aΚB2M TCGCGATGGCCCGGGAGTCGGGGACCTCGGCT, 34aΚB3M CCGTCGGTCTGGCGACAGCGCAGCTCCCCGGA) were added prior to the labelled probe. For supershift assays, antibodies against p65, p50 and p53 were added in the binding reaction after DNA-protein incubation. After electrophoresis, DNA-protein complex was transferred to a nylon membrane, and cross-linked. Then the biotinylated-labelled DNA detected by chemiluminescence according to the manufacturer's directions.

### Statistics

Statistical analyses were performed with SPSS 16.0 (SPSS Inc., Chicago, IL, USA). Differences between experimental groups and control groups were assessed by Student's t-test. P < 0.05 was considered to be statistically significant.

## List of abbreviations

CDK4/6: cyclin-dependent kinase 4/6; SIRT1: silent information regulator 1; MET: hepatocyte growth factor receptor; C/EBPα: CCAAT enhancer binding protein alpha; NF-κB: Nuclear factor-kappa B; IκB: nuclear factor of kappa light polypeptide gene enhancer in B-cells inhibitor.

## Authors' contributions

JL carried out the majority of the cellular and molecular studies, participated in drafted the manuscript. KW carried out western blot assays. XC participated in CHIP assays. HM participated in qRT-PCR assay. M S and YW participated in result analysis and helped to draft the manuscript. XX helped to do bioinformatic analysis, experiment design and result analysis. YB conceived of the study, and participated in its design and coordination and helped to draft the manuscript. All authors read and approved the final manuscript.
